# Examining the Association between Trauma Exposure and Work-Related Outcomes in Women Veterans

**DOI:** 10.3390/ijerph17124585

**Published:** 2020-06-25

**Authors:** Megan E. Sienkiewicz, Aneline Amalathas, Katherine M. Iverson, Brian N. Smith, Karen S. Mitchell

**Affiliations:** 1 National Center for PTSD Women’s Health Sciences Division, VA Boston Healthcare System, Boston, MA 02130, USA; megan.sienkiewicz@va.gov (M.E.S.); katherine.Iverson@va.gov (K.M.I.); Brian.Smith12@va.gov (B.N.S.); 2 Ochsner Clinical School, University of Queensland, New Orleans, LA 70121, USA; anelineamalathas@gmail.com; 3 Department of Psychiatry, Boston University School of Medicine, Boston, MA 02118, USA

**Keywords:** traumatic stress, military trauma, PTSD, depression, unemployment, occupational health, veterans’ health

## Abstract

Women veterans have high rates of trauma exposure, including military sexual trauma (MST), which are associated with numerous health and psychosocial consequences. However, associations between trauma history and work-related outcomes are less well-characterized. We examined whether military-related and non-military trauma types were associated with work-related outcomes and whether posttraumatic stress disorder (PTSD) and depression symptoms mediated these associations. A total of 369 women veterans completed up to two mailed surveys, 12 months apart, assessing trauma exposure, depression and PTSD symptoms, occupational functioning, and employment status (unemployed, out of the workforce, employed). Participants reported high rates of trauma exposure. Nearly half (47.5%) were out of the workforce. Military-related trauma, military sexual assault, and adult sexual assault were associated with worse occupational functioning. Only PTSD symptoms mediated associations between trauma types and occupational functioning. No trauma types were significantly directly associated with employment status; however, PTSD and depression symptoms mediated associations between trauma types and being out of the workforce. Findings can inform screening for military trauma exposures, mental health, and work-related needs among women veterans.

## 1. Introduction

The majority (51–69%) of women in the U.S. report trauma exposure at some point in their lives [1], including adult and childhood sexual assault and physical assault, and intimate partner violence (IPV). Women veterans experience particularly high rates of lifetime trauma exposure (i.e., 81–93%) [2], including military sexual trauma (MST) [3], defined as sexual harassment and/or sexual assault experiences during military service. The latest Department of Defense (DoD) annual report on sexual assault in the military [4] reported that 24.2% of active duty females experienced sexual harassment during the 2018 fiscal year and 6.2% experienced sexual assault during the same period. Women veterans also report high rates of interpersonal violence in general, e.g., 37% of a sample of women veterans reported past-year intimate partner violence (IPV) [5]. Further, an unprecedented number of women in the military are exposed to combat trauma, which has been linked to increased rates of posttraumatic stress disorder (PTSD), depression, and alcohol misuse [6,7]. In addition to physical and mental health consequences, trauma exposure can contribute to impaired occupational functioning among veterans [8], although this has been studied less frequently, particularly among women.

Relatively little is known about the relationship between trauma and work-related outcomes, including employment and occupational functioning. The majority of previous studies have focused on PTSD symptoms and diagnoses, rather than types of trauma exposure, and employment-related outcomes. Symptom severity of PTSD has been negatively associated with work-related quality of life among veterans [9,10]. Vogt and colleagues [11] found that women veterans with probable PTSD were more likely to report impaired work performance and nearly twice as likely than those without probable PTSD to report job dissatisfaction. Further, veterans with a lifetime diagnosis of PTSD were less likely to be currently working than veterans without PTSD and employed veterans with PTSD earned on average 16% less per hour than those without PTSD [12].

Of the studies examining the impact of trauma exposures specifically on work-related outcomes, one found that women exposed to interpersonal violence (e.g., assault) resulting in hospitalization were less often gainfully employed (30%) than women who were not exposed to such violence (65%) [13]. Women who experienced interpersonal violence, including rape, sexual assault, IPV, and stalking, reported greater levels of mental health-related dysfunction, disability, and impaired quality of life [14]. Recent sexual trauma (including sexual harassment and sexual assault) among female military personnel was associated with significantly higher odds of work functioning-related difficulties due to physical and mental health [15]. In a recent study of post-9/11 male and female veterans, physical, psychological, and sexual IPV experiences were associated with lower job satisfaction and employment functioning [16]. 

Several studies have examined the impact of military-specific trauma exposures, including warfare and MST, on occupational outcomes among male and female veterans. A recent study found that warfare exposure predicted worse occupational satisfaction for men and women [10]. In the same study, sexual harassment during military service had negative implications for work-related quality of life; this association was mediated by PTSD, depression, and alcohol misuse symptom severity [10]. 

Despite these findings, little is known about the impact of specific types of trauma exposure on work-related outcomes. Combat trauma or interpersonal violence, considered among the most severe and impactful exposures [17], may differentially impact employment and occupational functioning; however, their differential impact on employment and occupational functioning has not been examined. In addition, the contribution of childhood assault to work-related outcomes has received little attention in the literature. Childhood assault, including physical and sexual assault, has been associated with a wide range of negative outcomes including neurodevelopmental and psychosocial developmental delays, increased risk of deviant social behaviors, learning and attention difficulties, depression, suicide, substance abuse, and teen pregnancy [18]. It also may increase the risk for adult-onset trauma and has been linked to outcomes including, but not limited to, increased risk of stressful life events, low income, medical conditions, anxiety, PTSD symptoms, depression, suicidality, and alcohol abuse [18,19,20,21,22,23]. To the best of our knowledge, no direct associations between childhood assault and impaired occupational functioning have been found. However, PTSD, emotion regulation, and interpersonal problems in those with histories of childhood assault have been associated with impaired role functioning [20], suggesting that childhood assault might be indirectly associated with work-related outcomes. Additionally, childhood assault and its effects on employment status have been underexplored and relatively inconclusive. Zielinkski [23] reported that those physically assaulted during childhood were 140% more likely to be unemployed in adulthood compared to non-victims of childhood maltreatment; yet Sansone, Leung, and Wiederman [24] found no association between childhood physical assault and employment variables. One study found that childhood sexual assault was associated with an increased likelihood of being fired as well as an increased number of full-time positions throughout adulthood [24], while another noted no difference in unemployment rates between childhood sexual assault survivors and those with no past childhood maltreatment [23]. 

This study contributes to these gaps in the literature by examining the effects of multiple types of traumatic experiences on employment status and occupational functioning in a sample of women veterans, who report lower rates of employment and lower median salaries compared to their male counterparts [11]. We hypothesized that women with histories of trauma exposure would have poorer work-related outcomes compared to women without trauma histories. Considering female veterans’ high rates of trauma exposure and unemployment, we also hypothesized that these associations would be strongest for military-related traumas, compared to other adulthood and childhood trauma exposures. Given previous findings that trauma exposure may be indirectly associated with work-related outcomes [10,20], PTSD and depression symptoms were examined as possible mediating variables.

## 2. Materials and Methods

### 2.1. Participants

Data for the present study are derived from a previous study regarding IPV among female New England Veterans Health Administration patients [25]. The original study was planned as a cross-sectional, but was later expanded to assess mental health symptoms and functioning over time. To investigate outcomes over time, participants were administered two mail surveys 12 months apart in 2012 (Time 1; T1) and 2013 (Time 2; T2). The current study is a secondary analysis of these data. A potential participant pool of 700 female veterans was randomly selected using the Veterans Health Administration Corporate Data Warehouse. Of this sampling pool, 581 veterans with locatable addresses were sent a survey, and 369 (63.5%) responded to the T1 survey. Of the 369 T1 participants, 216 agreed to be re-contacted and 198 responded to a T2 follow-up survey (79.8% response rate). The majority of the 369 participants identified as White or Caucasian (83.5%); 2.4% were of Hispanic/Latino origin or descent, 4.3% were American Indian or Alaskan Native, 0.3% were Pacific Islander, 0.5% were Asian, 8.1% were African American or Black, and 4.1% identified as “other” race (categories are not mutually exclusive). Their mean age was 55.7 (*SD* = 17.2). 

### 2.2. Data Collection

Data were collected using a modified Dillman [26] multiple mailing strategy. At each time point, the survey procedures were as follows: (a) an informed consent fact sheet, survey instrument, and $10 cash incentive were mailed to potential participants; (b) two weeks later, a thank you postcard and reminder to respond were sent to all potential participants; and (c) four weeks later, another copy of the survey and $10 cash incentive were mailed to all potential participants who had not responded.

### 2.3. Measures—Time 1

#### 2.3.1. Trauma History Screen (THS)

The THS is a 14-item assessment of trauma exposure [27]. Respondents answer “Yes” or “No” to each potentially traumatic event. We included the following trauma exposures, coded 0/1: childhood physical assault, adult physical assault, childhood sexual assault, adult sexual assault, and military-related trauma (i.e., during military service, saw something horrible or was scared badly). In the development sample, the THS had high convergent validity when compared to other trauma screens [27]. 

#### 2.3.2. Military Sexual Trauma (MST) Screen

The MST Screen consists of two items used to screen for MST in Veterans Health Administration: “While you were in the military… Did you receive uninvited and unwanted sexual attention (e.g., touching, cornering, pressure for sexual favors or verbal remarks)? Did someone ever use force or threat of force to have sexual contact with you against your will?” Screening positive for MST using these items has been associated with higher rates of mental health conditions [28]. We examined sexual harassment and assault individually and also created a dichotomous variable indicating whether participants answered “Yes” to either of the two items. 

#### 2.3.3. Employment

Participants were asked to mark all applicable current employment options, including “Working for pay full-time (>30 h/week)”, “Working for pay part-time (<30 h/week)”, “Working as volunteer (no pay)”, “Student in high school, job training, or college degree program”, “Homemaker”, “Not working but actively looking for work”, “Not working and not looking for work”, “Retired”, and “Unable to work”. Participants who reported working full time or part time were classified as employed. Participants who reported being not employed but were looking for work were classified as unemployed. Participants who reported being unable to work, retired, or unemployed and not looking for work were considered out of the workforce. Participants who reported being a student, volunteer, or homemaker and did not also fall into one of the aforementioned categories were excluded from analyses. 

These employment categories are similar to those described by the U.S. Bureau of Labor Statistics [29], except that we were unable to separate individuals who were actively looking for work vs. “discouraged workers” who have looked for work in the past 12 months but not within the past 4 weeks. Further, unlike the Bureau of Labor Statistics, we excluded students and homemakers from the “out of the workforce” category in order to separate presumably higher functioning patients from those who are potentially unable to work due to disability, consistent with other recent studies [30,31]. 

#### 2.3.4. The Center for Epidemiologic Studies-Depression Scale (CES-D)

The CES-D [32] is a 20-item self-report measure of depression symptoms. Questions target depressive symptomatology components and lead with “How often in the past week…” to get an idea of the participant’s current state. Respondents choose from 4 responses, ranging from “Rarely or none of the time” to “Most or all of the time”. Frequency of symptom occurrence was measured from 0 to 3. Scores range from 0 to 60, with higher scores indicating more symptoms. Cronbach’s alpha in this study was 0.80.

#### 2.3.5. The Posttraumatic Stress Disorder Checklist (PCL)

The PCL is a self-report assessment of *Diagnostic and Statistical Manual of Mental Disorder-IV* [33] PTSD symptoms and consists of 17 items [34]. Respondents use a 5-point scale to report the level to which they have been bothered by each symptom in the past month. Possible responses range from “Not at all” to “Extremely”. Items are summed to create an overall severity score. Cronbach’s alpha in this study was 0.96.

Covariates: included Time 1 age and education. 

### 2.4. Measures—Time 2

Employment status was assessed again at Time 2.

#### Inventory of Psychosocial Functioning

The Inventory of Psychosocial Functioning (IPF) [35,36] is an assessment of potential impairments in functioning over multiple domains, such as family relationships and self-care. The IPF has exhibited high levels of convergent validity with other impairment measures [35,36]. We analyzed the 21 items that assess occupational health as measured by difficulties at work. Respondents who reported being employed (paid for full or part-time work or as a volunteer; *n* = 90) in the past 30 days were asked about functioning during this time frame. Response options to this items use a 7-point scale ranging from “Never” to “Always”. Scores range from 0 to 100; we recoded this measure so that higher scores indicate better occupational functioning. Cronbach’s alpha in this study was 0.75. 

### 2.5. Statistical Analyses

Descriptive statistics, using IBM SPSS Statistics 20 (IBM, Armonk, NY, USA), were used to calculate the proportion of participants who endorsed each trauma type and who were employed or unemployed. Regression models were estimated using Mplus 7.0 [37]. Each trauma type was included as an independent variable in separate regression models. Multiple linear regression models were estimated to investigate associations between trauma types and Time 2 occupational functioning. Multinomial logistic regression models were used to investigate associations between trauma types and a three-category employment variable (employed, unemployed, and out of the workforce at Time 2). We calculated McFadden’s R^2^ values, which reflect the level of improvement of the estimated model compared to the null model, for models with employment status as the dependent variable as 1-(loglikelihood of the estimated model/loglikelihood of the null model).

We examined the potential mediating roles of depression and PTSD symptoms, respectively, for all models as independent variables may be indirectly associated with outcomes, even in the absence of direct effects [38]. Time 1 PCL or CES-D scores, respectively, were used as mediators. In Mplus, mediation is typically investigated using bootstrap estimates to obtain confidence intervals for the indirect effect in models with continuous outcomes. This option is not available for nominal outcomes, however. Thus, for models with employment status as the dependent variable, we used the Model Constraint command to create the product term by multiplying the coefficients for the path from the independent variable to the mediator and from the mediator to the dependent variable. Bootstrapping was used to calculate a 95% confidence interval to test the significance of the product term [37].

In order to account for missing data, we examined which Time 1 variables were associated with participation in the Time 2 survey. Based on Time 1 data, we found that Time 2 participants were significantly younger (*T*_295_ = 3.68, *p* < 0.001) and more educated (*T*_357_ = −2.25, *p* = 0.03) compared to Time 1 only participants. In addition, 53.4% of participants who were out of the workforce at Time 1 did not complete the Time 2 survey (χ^2^ = 7.52, df = 1, *p* = 0.01). Time 1 PCL (*T*_333_ = −0.76, *p* = 0.45) and CES-D scores (*T*_248_ = −0.49, *p* = 0.63) were not associated with Time 2 participation. A higher proportion of Time 2 participants endorsed MST (χ^2^ = 4.98, df = 1, *p* = 0.03) and adulthood sexual assault (χ^2^ = 7.01, df = 1, *p* = 0.01) compared to Time 1 only participants. Therefore, we addressed missing data in the total sample by including Time 1 age and education as well as a dummy variable representing being out of the workforce in all models. For the linear regression models with occupational functioning as an outcome, we restricted the sample to the 90 respondents who completed the occupational functioning measure at Time 2 (as described in the Measures section) and then used full information maximum likelihood to analyze all available data. For the multinomial logistic regression models with employment status as the dependent variable, we included all participants and analyzed all available data via robust maximum likelihood.

### 2.6. Ethical Considerations

This study was approved by the International Review Board at VA Boston Healthcare System on 31 October 2011 (IRB #2606). All participants received an informed consent fact sheet with survey materials. Data can be shared via a Freedom of Information Act Request.

## 3. Results

In our sample, 76 participants were employed (38.4%), nine were unemployed (4.5%), and 93 were out of the workforce (47.5%). Rates of trauma exposure were as follows: childhood sexual assault (25.3%), childhood physical assault (17.2%), adult sexual assault (36.4%), adult physical assault (20.2%), military-related trauma (38.9%), and any MST (54.5%); 54.0% of women reported military sexual harassment (MSH) and 26.8% reported military sexual assault (MSA). 

See Table 1 for a summary of linear regression models assessing associations between military and non-military trauma types and occupational functioning. 

MSA, adult sexual assault, and military-related trauma were associated with occupational functioning, such that women who had experienced these traumas reported lower (worse) occupational functioning. 

See Table 2 for a summary of multinomial logistic regression models assessing associations between military and non-military trauma types and employment. 

No trauma types were directly associated with employment status. 

We also examined depression and PTSD symptoms as mediators of the associations between military-related and non-military related traumas and occupational functioning and employment status, respectively. Due to the high correlation between CESD-D scores and PCL scores (*r* = 0.83), we analyzed these variables in separate models. See Table 3 and Table 4 for full mediation model results with employment status as the outcome.

There were no significant indirect effects between trauma type and being unemployed with PTSD or depression symptoms as the mediator. PTSD symptom scores mediated the relationships between MST, MSA, MSH, military-related trauma, adult physical assault, adult sexual assault, childhood physical assault, and childhood sexual assault, respectively, and being out of the workforce. Similarly, depression symptom scores were found to mediate the relationships between MST, MSA, MSH, military-related trauma, adult physical assault, and adult sexual assault and being out of the workforce. This suggests that both PTSD and depression symptoms may partly account for the associations between trauma and being out of the work force, relative to being employed.

See Supplementary Tables S1 and S2 for full mediation model results with occupational functioning as the outcome. We found that PTSD symptom scores mediated the relationships between MST, MSA, MSH, military-related trauma, adult physical assault, adult sexual assault, and childhood physical assault, respectively, and occupational functioning. This suggests that PTSD symptoms may partly account for the associations between various trauma types and occupational functioning. CES-D scores did not mediate the associations between trauma type and occupational functioning (all *p* < 0.05). 

## 4. Discussion

This study examined the relationship between trauma exposure, employment, and occupational functioning in women veterans. We anticipated that occupational functioning would be worse among employed women with trauma histories. Results supported this hypothesis, with military-related trauma (not including MST), military sexual assault, and adult sexual assault being significantly associated with worse occupational functioning. For military-related trauma, this could in part be due to the fact that combat exposure may tap into existing vulnerabilities and subsequently affect psychosocial functioning broadly [39]. Considering that military-related traumas occurred in the context of one’s work duties as part of military service, it is possible that such exposures are particularly likely to impact work functioning. Additionally, military-related trauma, including combat, may be a particularly severe form of trauma, resulting in higher rates of negative outcomes, including PTSD, psychosocial impairment, and poorer physical health [40,41].

Our finding that MSA and adult sexual assault were significantly associated with worse occupational functioning highlights the impact of adulthood sexual trauma on work-related outcomes among women veterans, regardless of whether it occurred during or outside of their military service. Notably, we found that MSH was not significantly associated with occupational functioning. This contributes to the few previous studies that found MSA to have worse outcomes in comparison to MSH [42,43,44]. MSA has been associated with depression diagnoses, probable sexual dysfunction, suicide ideation, and probable PTSD, while MSH showed no such associations [42]. In a study of male and female veterans, participants reporting MSA had a greater risk of lifetime and current PTSD compared to participants reporting only MSH [44]. In a separate sample of female veterans, MSA was associated with PTSD symptoms while MSH was not [43]. These results emphasize the importance of separately analyzing the effects of MSH and MSA. To our knowledge, our study was the first to investigate the differential impact of MSA and MSH on occupational outcomes. Further research is needed to confirm the current finding and explicate possible mechanisms of this association.

We also anticipated that women with trauma exposure histories would be less likely to be employed or out of the workforce. Our findings partially supported this hypothesis. We found no significant direct or indirect associations between trauma types and being unemployed relative to employed. This was somewhat surprising given past literature having found interpersonal trauma, mental health conditions, and gender to inhibit female veteran civilian employment [9,11,31,45,46]. However, as hypothesized, we found that PTSD and depression symptoms mediated associations between trauma types and employment outcomes. The majority of trauma types were indirectly associated with being out of the workforce, e.g., being retired or unable to work due to physical or mental disability, through both PTSD and depression symptoms. Thus, our findings illustrate the impact of interpersonal trauma-related sequelae on employment among female veterans. 

Only PTSD symptoms significantly mediated associations between all trauma types (except childhood sexual assault) and occupational functioning. This supports findings from previous studies showing associations between PTSD symptoms and work-related quality of life and impaired work performance [9,10,11,15,16,39]. Our findings underscore the importance of addressing both military and non-military related interpersonal trauma sequalae as early as possible, given findings that rates of PTSD and serious functional impairment among Army soldiers and National Guard soldiers increased from 3 months post deployment to 12 months post deployment [47].

In contrast, we did not find evidence that depression mediated associations between trauma types and occupational functioning. Examination of R^2^ values suggests that associations between most forms of trauma exposure and depression symptoms were weaker than associations between trauma and PTSD symptoms. Thus, our study may have been underpowered to detect the weaker effects of the indirect effect of trauma on occupational functioning through depression. Consistent with our findings, a previous study among National Guard/Reserve veterans found that PTSD, but not depression and alcohol-related diagnoses, was significantly associated with a decrease in work/school role functioning over time [48].

Our findings should be interpreted in light of the study’s limitations. Our wide confidence intervals suggest that low power may have impacted our findings. In addition, lack of differentiation between chronic or isolated trauma exposures, including potential overlap between adult sexual trauma and military sexual trauma, may have contributed to the lack of significant direct effects in the associations between specific trauma types and work-related outcomes. Our study was further limited by the measurement of employment status. In particular, the out of the workforce category was quite heterogeneous. Although we excluded participants who reported being a student, homemaker, or volunteer from this category, we do not know participants’ reasons for retirement or why they were unemployed but not looking for work. Thus, although we assume that many participants in this category are not working due to impairment, we could not determine this. We also do not have data on service-connected disabilities, which may play a role in the associations observed and could be examined in future research. Our findings were based on a primarily Caucasian, women veteran sample, therefore it is unclear how well results would generalize to male veterans, non-veterans, or to more ethnically/racially diverse individuals. 

## 5. Conclusions

Our findings have implications for improving health care and employment-related outcomes for women following military service. Early detection and intervention for PTSD and depression symptoms following both military-related and non-military-related traumas, with an emphasis on military and non-military sexual trauma and combat exposure, could help reduce the long-term consequences of such experiences on women’s psychosocial functioning. Increased provider awareness of the links between these exposures could inform enhanced inquiry regarding trauma exposures and assessment of related mental health symptoms. 

This study provides new knowledge regarding trauma exposure, employment, and occupational functioning in women veterans. Future literature must consider how mental health outcomes following military and non-military trauma impacts the likelihood of being out of the workforce due to circumstances such as early retirement or disability. Regarding MST specifically, future work in this area should take into consideration and expand upon potential differing effects of MSA and MSH on work-related outcomes. Ultimately, further research on this topic is required to better understand the impact of trauma exposure on occupational functioning and employment, particularly for women veterans, who are more likely to be adversely affected by trauma, PTSD, and depression in this domain of functioning. 

## Figures and Tables

**Table 1:** Associations between trauma exposures and occupational functioning.

Independent Variable	Occupational Functioning					
	B	β	SE	*p*	R^2^	95% CI
Military sexual trauma	−2.95	−0.12	2.51	0.24	0.07	−7.87, 1.98
Military sexual assault	−6.56	−0.24	2.82	0.02 *	0.12	−12.08, −1.04
Military sexual harassment	−3.29	−0.14	2.55	0.20	0.08	−8.29, 1.71
Military-related trauma	−7.01	−0.29 *	2.55	0.01 *	0.14	−12.01, −2.00
Adult physical assault	−4.43	−0.14	3.21	0.17	0.07	−10.72, 1.85
Adult sexual assault	−6.93	−0.27	2.58	0.01 *	0.13	−11.99, −1.87
Childhood physical assault	−4.33	−0.14	3.12	0.17	0.07	−10.45, 1.79
Childhood sexual assault	−2.03	−0.07	3.13	0.52	0.05	−8.16, 4.10

Note: SE = standard error, CI = confidence interval. Higher occupational functioning scores indicate better outcomes. All models controlled for Time 1 age, education, and employment status. *n* = 90 because only women who reported being employed or volunteering in the past 30 days completed the occupational functioning measure and were included in the analyses. * *p* < 0.05.

**Table 2:** Associations between trauma exposures and employment status.

Path	B	β	SE	*p*	*R^2^*	OR	95% CI
Unemployed							
MST → Unemployed	−0.09	−0.07	0.76	0.91	0.03	0.91	−1.57, 1.39
MSA → Unemployed	−0.43	−0.28	1.18	0.71	0.08	0.65	−2.75, 1.89
MSH → Unemployed	−0.06	−0.05	0.75	0.94	0.03	0.94	−1.53, 1.41
MRT → Unemployed	−0.22	−0.16	0.90	0.81	0.03	0.80	−198, 1.54
APA → Unemployed	1.68	0.70	0.85	0.05	0.03	5.35	0.002, 3.35
ASA → Unemployed	−0.53	−0.33	0.83	0.52	0.03	0.59	−2.16, 1.10
CPA → Unemployed	0.86	0.46	0.75	0.25	0.03	2.36	−0.60, 2.32
CSA → Unemployed ^a^	--	--	--	--	--	--	--
Out of the Work Force (OOWF)							
MST → OOWF	0.23	0.04	0.58	0.70	0.03 ^b^	1.26	−0.92, 1.37
MSA → OOWF	0.64	0.10	0.66	0.33	0.08 ^b^	1.91	−0.65, 1.94
MSH → OOWF	0.29	0.05	0.60	0.63	0.03 ^b^	1.33	−0.88, 1.46
MRT → OOWF	−0.1	−0.002	0.62	0.99	0.03 ^b^	0.99	−1.22, 1.19
APA → OOWF	0.53	0.07	0.60	0.38	0.03 ^b^	1.70	−0.64, 1.70
ASA → OOWF	−0.04	−0.01	0.69	0.95	0.03 ^b^	0.96	−1.40, 1.32
CPA → OOWF	−0.77	−0.11	0.87	0.38	0.03 ^b^	0.46	−2.47, 0.94
CSA → OOWF	0.14	0.02	0.78	0.86	0.03 ^b^	1.15	−1.39, 1.67

Note: MST = military sexual trauma, MSA = military sexual assault, MSH = military sexual harassment, MRT = military-related trauma, APA = adult physical assault, ASA = adult sexual assault, CPA = child physical abuse, CSA = child sexual abuse, SE = standard error, OR = odds ratio, CI = confidence interval. ORs reflect the likelihood of the outcome for no exposure vs. exposure. Betas and ORs reflect the odds of being unemployed or out of the workforce, respectively, relative to being employed. Trauma exposure was assessed at Time 1; employment status was assessed at Time 2. Models are adjusted for Time 1 age, education, and employment status. Nagelkerke’s *R*^2^ is reported for logistic regression models. ^a^ no participants reporting a history of childhood sexual abuse were classified as unemployed. ^b^ McFadden’s *R*^2^ values are reported for the overall model with the 3-category employment status (employed, unemployed, and out of the workforce) variable as the dependent variable. Therefore, McFadden’s *R*^2^ values are identical for the unemployed and out of the workforce categories.

**Table 3:** Associations between trauma exposure and employment status, with depression symptoms as a mediator.

Path	B	β	SE	*p*	*R^2^*	OR	95% CI
Military Sexual Trauma (MST)							
Unemployed							
MST → CESD → Unemployed							
MST → CESD (a)	6.04	0.31	1.09	<0.001	0.09	--	3.90, 8.18
CESD → Unemployed (b)	0.08	0.77	0.04	0.06	--	1.08	−0.004, 0.16
MST → Unemployed (c)	−0.09	−0.07	0.76	0.91	0.03	0.91	−1.57, 1.39
MST → CESD → Unemployed (ab)	0.47	--	0.27	0.08	--	--	−0.05, 0.99
MST → Unemployed with CESD (c’)	−0.60	−0.30	0.88	0.49	0.02	0.55	−2.32, 1.12
Out of the Workforce (OOWF)							
MST → CESD → OOWF							
MST → CESD (a)	6.04	0.31	1.09	<0.001	0.09	--	3.90, 8.18
CESD → OOWF (b)	0.12	0.35	0.04	0.01	--	1.12	0.03, 0.20
MST → OOWF (c)	0.23	0.04	0.58	0.70	0.03 ^b^	1.26	−0.92, 1.37
MST → CESD → OOWF (ab)	0.7	--	0.31	0.02	--	--	0.10, 1.30
MST → OOWF with CESD (c’)	−0.39	−0.06	0.56	0.49	0.02 ^b^	0.68	−1.48, 0.70
Military Sexual Assault (MSA)							
Unemployed							
MSA → CESD → Unemployed							
MSA → CESD (a)	6.59	0.30	1.34	<0.001	0.09	--	3.97, 9.21
CESD → Unemployed (b)	0.09	0.77	0.04	0.04	--	1.09	0.004, 0.17
MSA → Unemployed (c)	−0.43	−0.28	1.18	0.71	0.08	0.65	−2.75, 1.89
MSA → CESD → Unemployed (ab)	0.56	--	0.29	0.05	--	--	−0.01, 1.13
MSA → Unemployed with CESD (c’)	−1.22	−0.50	1.54	0.43	0.02	0.29	−4.24, 1.79
Out of the Workforce (OOWF)							
MSA → CESD → OOWF							
MSA → CESD (a)	6.59	0.30	1.34	<0.001	0.09	--	3.97, 9.21
CESD → OOWF (b)	0.12	0.34	0.05	0.01	--	1.12	0.03, 0.21
MSA → OOWF (c)	0.64	0.10	0.66	0.33	0.08 ^b^	1.91	−0.65, 1.94
MSA → CESD → OOWF (ab)	0.76	--	0.35	0.03	--	--	0.08, 1.44
MSA → OOWF with CESD (c’)	0.01	0.001	0.70	0.99	0.02 ^b^	1.01	−1.35, 1.37
Military Sexual Harassment (MSH)							
Unemployed							
MSH → CESD → Unemployed							
MSH → CESD (a)	6.16	0.31	1.08	<0.001	0.10	--	4.05, 8.27
CESD → Unemployed (b)	0.08	0.78	0.04	0.07	--	1.08	−0.01, 0.16
MSH → Unemployed (c)	−0.06	−0.05	0.75	0.94	0.03	0.94	−1.53, 1.41
MSH → CESD → Unemployed (ab)	0.48	--	0.28	0.08	--	--	−0.06, 1.02
MSH → Unemployed with CESD (c’)	−0.58	−0.29	0.88	0.51	0.02	0.56	−2.30, 1.14
Out of the Workforce (OOWF)							
MSH → CESD → OOWF							
MSH → CESD (a)	6.16	0.31	1.08	<0.001	0.10	--	4.05, 8.27
CESD → OOWF (b)	0.12	0.35	0.05	0.01	--	1.12	0.03, 0.20
MSH → OOWF (c)	0.29	0.05	0.60	0.63	0.03 ^b^	1.33	−0.88, 1.46
MSH → CESD → OOWF (ab)	0.72	--	0.31	0.02	--	--	0.11, 1.33
MSH → OOWF with CESD (c’)	−0.36	−0.06	0.56	0.52	0.02 ^b^	0.70	−1.47, 0.74
Military Related Trauma (MRT)							
Unemployed							
MRT → CESD → Unemployed							
MRT → CESD (a)	8.94	0.44	1.06	<0.001	0.20	--	6.87, 11.02
CESD → Unemployed (b)	0.09	0.84	0.04	0.05	--	1.09	0.000, 0.17
MRT → Unemployed (c)	−0.22	−0.16	0.90	0.81	0.03	0.80	−1.98, 1.54
MRT → CESD → Unemployed (ab)	0.77	--	0.40	0.06	--	--	−0.02, 1.55
MRT → Unemployed with CESD (c’)	−0.98	−0.48	0.97	0.31	0.02	0.38	−2.87, 0.91
Out of the Workforce (OOWF)							
MRT → CESD → OOWF							
MRT → CESD (a)	8.94	0.44	1.06	<0.001	0.20	--	6.87, 11.02
CESD → OOWF (b)	0.14	0.39	0.06	0.03	--	1.14	0.02, 0.25
MRT → OOWF (c)	−0.1	−0.002	0.62	0.99	0.03 ^b^	0.99	−1.22, 1.19
MRT → CESD → OOWF (ab)	1.21	--	0.58	0.04	--	--	0.07, 2.34
MRT → OOWF with CESD (c’)	−1.05	−0.15	0.82	0.20	0.02 ^b^	0.35	−2.65, 0.55
Adult Physical Assault (APA)							
Unemployed							
APA → CESD → Unemployed							
APA → CESD (a)	9.98	0.05	1.30	<0.001	0.16	--	7.42, 12.55
CESD → Unemployed (b)	0.05	0.34	0.04	0.20	--	1.05	−0.03, 0.12
APA → Unemployed (c)	1.68	0.70	0.85	0.05	0.03	5.35	0.002, 3.35
APA → CESD → Unemployed (ab)	0.46	--	0.36	0.21	--	--	−0.25, 1,17
APA → Unemployed with CESD (c’)	1.30	0.25	0.89	0.14	0.02	3.68	−0.43, 3.04
Out of the Workforce (OOWF)							
APA → CESD → OOWF							
APA → CESD (a)	9.98	0.05	1.30	<0.001	0.16	--	7.42, 12.55
CESD → OOWF (b)	0.12	0.11	0.05	0.03	--	1.11	0.01, 0.20
APA → OOWF (c)	0.53	0.07	0.60	0.38	0.03 ^b^	1.70	−0.64, 1.70
APA → CESD → OOWF (ab)	1.07	--	0.51	0.03	--	--	0.08, 2.06
APA → OOWF with CESD (c’)	−0.23	0.07	0.58	0.70	0.02 ^b^	0.80	−1.37, 0.91
Adult Sexual Assault (ASA)							
Unemployed							
ASA → CESD → Unemployed							
ASA → CESD (a)	6.99	0.33	1.19	<0.001	0.11	--	4.65, 9.32
CESD → Unemployed (b)	0.08	0.77	0.04	0.05	--	1.09	0.002, 0.17
ASA → Unemployed (c)	−0.53	−0.33	0.83	0.52	0.03	0.59	−2.16, 1.10
ASA → CESD → Unemployed (ab)	0.59	--	0.31	0.05	--	--	−0.01, 1.18
ASA → Unemployed with CESD (c’)	−1.18	−0.51	1.02	0.25	0.02	0.31	−3.17, 0.82
Out of the Workforce (OOWF)							
ASA → CESD → OOWF							
ASA → CESD (a)	6.99	0.33	1.19	<0.001	0.11	--	4.65, 9.32
CESD → OOWF (b)	0.11	0.33	0.05	0.02	--	1.12	0.01, 0.21
ASA → OOWF (c)	−0.04	−0.01	0.69	0.95	0.03 ^b^	0.96	−1.40, 1.32
ASA → CESD → OOWF (ab)	0.77	--	0.37	0.04	--	--	0.05, 1.48
ASA → OOWF with CESD (c’)	−0.64	−0.09	0.68	0.35	0.02 ^b^	0.53	−1.97, 0.69
Child Physical Assault (CPA)							
Unemployed							
CPA → CESD → Unemployed							
CPA → CESD (a)	4.36	0.17	1.33	0.001	0.03	--	1.75, 6.97
CESD → Unemployed (b)	0.06	0.65	0.04	0.11	--	1.06	−0.01, 0.14
CPA → Unemployed (c)	0.86	0.46	0.75	0.25	0.03	2.36	−0.60, 2.32
CPA → CESD → Unemployed (ab)	0.27	--	0.19	0.15	--	--	−0.10, 0.64
CPA → Unemployed with CESD (c’)	0.58	0.24	0.79	0.47	0.02	1.79	−0.98, 2.14
Out of the Workforce (OOWF)							
CPA → CESD → OOWF							
CPA → CESD (a)	4.36	0.17	1.33	0.001	0.03	--	1.75,6.97
CESD → OOWF (b)	0.12	0.34	0.05	0.02	--	1.13	0.02, 0.22
CPA → OOWF (c)	−0.77	−0.11	0.87	0.38	0.03 ^b^	0.46	−2.47, 0.94
CPA → CESD → OOWF (ab)	0.52	--	0.28	0.06	--	--	−0.03, 1.06
CPA → OOWF with CESD (c’)	−1.28	−0.15	0.81	0.11	0.02 ^b^	0.29	−2.86, 0.30
Child Sexual Assault (CSA)							
Unemployed							
CSA → CESD → Unemployed							
CSA → CESD (a)	4.32	0.19	1.28	0.001	0.04	--	1.81, 6.82
CESD → Unemployed (b)	0.07	0.32	0.04	0.09	--	1.07	−0.01, 0.15
CSA → Unemployed (c) ^a^	--	--	--	--	--	--	--
CSA → CESD → Unemployed (ab) ^a^	--	--	--	--	--	--	--
CSA → Unemployed with CESD (c’) ^a^	--	--	--	--	--	--	--
Out of the Workforce (OOWF)							
CSA → CESD → OOWF							
CSA → CESD (a)	4.32	0.19	1.28	0.001	0.04	--	1.81, 6.82
CESD → OOWF (b)	0.11	0.32	0.05	0.03	--	1.11	0.01, 0.20
CSA → OOWF (c)	0.14	0.02	0.78	0.86	0.03	1.15	−1.39, 1.67
CSA → CESD → OOWF (ab)	0.46	--	0.26	0.08	--	--	−0.05, 0.97
CSA → OOWF with CESD (c’)	−0.22	−0.03	0.82	0.79	0.02	0.81	−1.82, 1.39

Note: CESD = Center for Epidemiological Studies-Depression Scale, SE = standard error, OR = odds ratio, CI = Confidence Interval. a = direct path from the trauma type to the mediator, b = direct path from the mediator to unemployment status, c = direct path from the trauma type to unemployment status, ab = indirect effect from the trauma type to unemployment status via the mediator, c’ = direct path from trauma type to unemployment status with the mediator in the model. Trauma exposure and depression symptoms were assessed at Time 1; employment status was assessed at Time 2. Models are adjusted for Time 1 age, education, and employment status. ^a^ no participants reporting a history of childhood sexual abuse were classified as unemployed. ^b^ McFadden’s R^2^ values are reported for the overall model with the 3-category employment status (employed, unemployed, and out of the workforce) variable as the dependent variable. Therefore, McFadden’s R^2^ values are identical for the unemployed and out of the workforce categories.

**Table 4:** Associations between trauma types and employment status, with PTSD symptoms as a mediator.

Path	B	β	SE	*p*	*R^2^*	OR	95% CI
Military Sexual Trauma (MST)							
Unemployed							
MST → PCL → Unemployed							
MST → PCL (a)	13.35	0.38	1.82	<0.001	0.14	--	9.78, 16.91
PCL → Unemployed (b)	0.05	0.79	0.03	0.13	--	1.05	−0.01, 0.10
MST → Unemployment (c)	−0.09	−0.07	0.76	0.91	0.03	0.91	−1.57, 1.39
MST → PCL → Unemployed (ab)	0.60	--	0.40	0.14	--	--	−0.19, 1.38
MST → Unemployed with PCL (c’)	−0.58	−0.29	0.86	0.50	0.02	0.56	−2.27, 1.10
Out of the Workforce (OOWF)							
MST → PCL → OOWF							
MST → PCL (a)	13.35	0.38	1.82	<0.001	0.14	--	9.78, 16.91
PCL → OOWF (b)	0.06	0.35	0.02	0.004	--	1.06	0.02, 0.10
MST → OOWF (c)	0.23	0.04	0.58	0.70	0.03 ^b^	1.26	−0.92, 1.37
MST → PCL → OOWF (ab)	0.81	--	0.31	0.01	--	--	0.21, 1.42
MST → OOWF with PCL (c’)	−0.56	−0.09	0.71	0.43	0.02 ^b^	0.57	−1.94, 0.83
Military Sexual Assault (MSA)							
Unemployed							
MSA → PCL → Unemployed							
MSA → PCL (a)	16.23	0.40	2.32	<0.001	0.16	--	11.69, 20.77
PCL → Unemployed (b)	0.05	0.83	0.03	0.05	--	1.06	−0.001, 0.11
MSA → Unemployment (c)	−0.43	−0.28	1.18	0.71	0.07 ^b^	0.65	−2.75, 1.89
MSA → PCL → Unemployed (ab)	0.87	--	0.46	0.06	--	--	−0.04, 1.78
MSA → Unemployed with PCL (c’)	−1.48	−0.57	1.27	0.24	0.02 ^b^	0.23	−3.96, 1.01
Out of the Workforce (OOWF)							
MSA → PCL → OOWF							
MSA → PCL (a)	16.23	0.40	2.32	<0.001	0.16	--	11.69, 20.77
PCL → OOWF (b)	0.05	0.31	0.02	0.02	--	1.56	0.01, 0.10
MSA → OOWF (c)	0.64	0.10	0.66	0.33	0.07 ^b^	1.91	−0.65, 1.94
MSA → PCL → OOWF (ab)	0.88	--	0.39	0.03	--	--	0.11, 1.64
MSA → OOWF with PCL (c’)	−0.07	−0.01	0.90	0.94	0.02 ^b^	0.94	−1.83, 1.70
Military Sexual Harassment (MSH)							
Unemployed							
MSH → PCL → Unemployed							
MSH → PCL (a)	13.23	0.37	1.83	<0.001	0.14	--	9.65, 16.81
PCL → Unemployed (b)	0.04	0.79	0.03	0.13	--	1.05	−0.01, 0.10
MSH → Unemployment (c)	−0.06	−0.05	0.75	0.94	0.03	0.94	−1.53, 1.41
MSH → PCL → Unemployed (ab)	0.58	--	0.40	0.14	--	--	−0.19, 1.36
MSH → Unemployed with PCL (c’)	−0.55	−0.28	0.86	0.52	0.02	0.58	−2.23, 1.13
Out of the Workforce (OOWF)							
MSH → PCL → OOWF							
MSH → PCL (a)	13.23	0.37	1.83	<0.001	0.14	--	9.65, 16.81
PCL → OOWF (b)	0.06	0.34	0.02	0.005	--	1.06	0.02, 0.10
MSH → OOWF (c)	0.29	0.05	0.60	0.63	0.03 ^b^	1.33	−0.88, 1.46
MSH → PCL → OOWF (ab)	0.79	--	0.31	0.01	--	--	0.19, 1.40
MSH → OOWF with PCL (c’)	−0.52	−0.08	0.72	0.48	0.02 ^b^	0.60	−1.94, 0.90
Military Related Trauma (MRT)							
Unemployed							
MRT → PCL → Unemployed							
MRT → PCL (a)	18.60	0.51	1.92	<0.001	0.26	--	14.84, 22.36
PCL → Unemployed (b)	0.05	0.92	0.04	0.16	--	1.05	−0.02, 0.13
MRT → Unemployment (c)	−0.22	−0.16	0.90	0.81	0.03	0.80	−1.98, 1.54
MRT → PCL → Unemployed (ab)	0.98	--	0.70	0.16	--	--	−0.40, 2.36
MRT → Unemployed with PCL (c’)	−1.20	−0.58	1.23	0.33	0.02	0.30	−3.61, 1.20
Out of the Workforce (OOWF)							
MRT → PCL → OOWF							
MRT → PCL (a)	18.60	0.51	1.92	<0.001	0.26	--	14.84, 22.36
PCL → OOWF (b)	0.07	0.40	0.02	0.001	--	0.27	0.03, 0.12
MRT → OOWF (c)	−0.1	0.002	0.62	0.99	0.03 ^b^	0.99	−1.22, 1.19
MRT → PCL → OOWF (ab)	1.33	--	0.44	0.002	--	--	0.47, 2.19
MRT → OOWF with PCL (c’)	−1.29	−0.20	0.74	0.08	0.02 ^b^	1.07	−2.75, 0.16
Adult Physical Assault (APA)							
Unemployed							
APA → PCL → Unemployed							
APA → PCL (a)	20.31	0.46	2.37	<0.001	0.21	--	15.66, 24.96
PCL → Unemployed (b)	0.02	0.41	0.02	0.34	--	1.02	−0.02, 0.07
APA → Unemployment (c)	1.68	0.70	0.85	0.05	0.03	5.35	0.002, 3.35
APA → PCL → Unemployed (ab)	0.47	--	0.49	0.34	--	--	−0.50, 1.44
APA → Unemployed with PCL (c’)	1.26	0.50	0.81	0.12	0.02	3.54	−0.32, 2.85
Out of the Workforce (OOWF)							
APA → PCL → OOWF							
APA → PCL (a)	20.31	0.46	2.37	<0.001	0.21	--	15.66, 24.96
PCL → OOWF (b)	0.06	0.32	0.02	0.01	--	1.06	0.01, 0.10
APA → OOWF (c)	0.53	0.07	0.60	0.38	0.03 ^b^	1.70	−0.64, 1.70
APA → PCL → OOWF (ab)	1.13	--	0.46	0.02	--	--	0.22, 2.04
APA → OOWF with PCL (c’)	−0.38	−0.05	0.73	0.60	0.02 ^b^	0.68	−1.81, 1.05
Adult Sexual Assault (ASA)							
Unemployed							
ASA → PCL → Unemployed							
ASA → PCL (a)	17.63	0.46	2.03	<0.001	0.21	--	13.66, 21.61
PCL → Unemployed (b)	0.06	0.88	0.03	0.04	--	1.07	0.003, 0.12
ASA → Unemployment (c)	−0.53	−0.33	0.83	0.52	0.03 ^b^	0.59	−2.16, 1.10
ASA → PCL → Unemployed (ab)	1.11	--	0.56	0.05	--	--	0.02, 2.20
ASA → Unemployed with PCL (c’)	−1.77	−0.64	0.94	0.06	0.02 ^b^	0.17	−3.62, 0.07
Out of the Workforce (OOWF)							
ASA → PCL → OOWF							
ASA → PCL (a)	17.63	0.46	2.03	<0.001	0.21	--	13.66, 21.61
PCL → OOWF (b)	0.07	−0.16	0.03	0.01	--	1.07	0.01, 0.13
ASA → OOWF (c)	−0.04	−0.01	0.69	0.95	0.03 ^b^	0.96	−1.40, 1.32
ASA → PCL → OOWF (ab)	1.22	--	0.53	0.02	--	--	0.18, 2.27
ASA → OOWF with PCL (c’)	−1.11	0.38	1.06	0.29	0.02 ^b^	0.33	−3.19, 0.96
Child Physical Assault (CPA)							
Unemployed							
CPA → PCL → Unemployed							
CPA → PCL (a)	12.63	0.28	2.52	<0.001	0.08	--	7.69, 17.56
PCL → Unemployed (b)	0.03	0.63	0.03	0.18	--	1.03	−0.02, 0.08
CPA → Unemployment (c)	0.86	0.46	0.75	0.25	0.03	2.36	−0.60, 2.32
CPA → PCL → Unemployed (ab)	0.42	--	0.33	0.21	--	--	−0.23, 1.07
CPA → Unemployed with PCL (c’)	0.55	0.23	0.80	0.49	0.02	1.73	−1.02, 2.11
Out of the Workforce (OOWF)							
CPA → PCL → OOWF							
CPA → PCL (a)	12.63	0.28	2.52	<0.001	0.08	--	7.69, 17.56
PCL → OOWF (b)	0.06	0.34	0.02	0.09	--	1.06	0.03, 0.10
CPA → OOWF (c)	−0.77	−0.11	0.87	0.38	0.03 ^b^	0.46	−2.47, 0.94
CPA → PCL → OOWF (ab)	0.77	--	0.28	0.01	--	--	0.22, 1.33
CPA → OOWF with PCL (c’)	−1.51	−0.18	0.90	0.09	0.02 ^b^	0.22	−3.27, 0.25
Child Sexual Assault CSA)							
Unemployed							
CSA → PCL → Unemployed							
CSA → PCL (a)	11.05	0.27	2.24	<0.001	0.07	--	6.65, 15.45
PCL → Unemployed (b)	0.04	0.33	0.03	0.12	--	1.04	−0.01, 0.09
CSA → Unemployment (c) ^a^	--	--	--	--	--	--	--
CSA → PCL → Unemployed (ab) ^a^	--	--	--	--	--	--	--
CSA → Unemployed with PCL (c’) ^a^	--	--	--	--	--	--	--
Out of the Workforce (OOWF)							
CSA → PCL → OOWF							
CSA → PCL (a)	11.05	0.27	2.24	<0.001	0.07	--	6.65, 15.45
PCL → OOWF (b)	0.05	0.31	0.02	0.003	--	1.06	0.02, 0.09
CSA → OOWF (c)	0.14	0.02	0.78	0.86	0.03	1.15	−1.39, 1.67
CSA → PCL → OOWF (ab)	0.60	--	0.24	0.01	--	--	0.13, 1.06
CSA → OOWF with PCL (c’)	−0.15	−0.02	0.81	0.85	0.02	0.86	−1.74, 1.43

Note: PCL = PTSD Check List, SE = standard error, OR = Odds Ratio, CI = Confidence Interval. a = direct path from trauma type to the mediator, b = direct path from the mediator to unemployment status, c = direct path from trauma type to unemployment status, ab = indirect effect from trauma type to unemployment status via the mediator, c’ = direct path from trauma type to unemployment status with the mediator in the model. Trauma exposure and PTSD symptoms were assessed at Time 1; employment status was assessed at Time 2. Models are adjusted for Time 1 age, education, and employment status. ^a^ no participants reporting a history of childhood sexual abuse were classified as unemployed. ^b^ McFadden’s R^2^ values are reported for the overall model with the 3-category employment status (employed, unemployed, and out of the workforce) variable as the dependent variable. Therefore, McFadden’s *R*^2^ values are identical for the unemployed and out of the workforce categories.

## Supplementary Materials

The following are available online at https://www.mdpi.com/1660-4601/17/12/4585/s1, Table S1: Associations between trauma types and occupational functioning, with depression symptoms as a mediator, Table S2: Associations between trauma types and occupational functioning, with PTSD symptoms as a mediator.
