# Supplementary material for: Examining the Association between Trauma Exposure and Work-Related Outcomes in Women Veterans
Source: Int J Environ Res Public Health. 2020 Jun 25;17(12):4585. doi: 10.3390/ijerph17124585 (PMC7344422; doi:10.3390/ijerph17124585)
Supplement: Supplementary file 1 [file ijerph-17-04585-s001.pdf]

International Journal of Environmental Research and Public Health

MDPI

Supplementary Materials

Table S1. Associations between trauma types and occupational functioning, with depression symptoms as a mediator.

Path	B	β	SE	*p*	R^2	95% CI
**Military Sexual Trauma (MST)**						
MST → CESD → Occ Fx						
MST → CESD (a)	5.51	0.36	1.63	0.001	0.13	2.32, 8.70
CESD → Occ Fx (b)	-0.39	-0.25	0.17	0.02	--	-0.71, -0.06
MST → Occ Fx (c)	-2.95	-0.12	2.51	0.24	0.07	-7.87, 1.98
MST → CESD → Occ Fx (ab)	-2.12	-0.09	1.17	0.07	--	-4.42, 0.18
MST → Occ Fx with CESD (c’)	-1.13	-0.05	2.80	0.69	0.13	-6.62, 4.37
**Military Sexual Assault (MSA)**						
MSA → CESD → Occ Fx						
MSA → CESD (a)	7.93	0.44	2.33	0.001	0.20	3.36, 12.50
CESD → Occ Fx (b)	-0.28	-0.18	0.19	0.11	--	-0.63, 0.07
MSA → Occ Fx (c)	-6.56	-0.24	2.82	0.02	0.12	-12.08, -1.04
MSA → CESD → Occ Fx (ab)	-2.25	-0.08	1.68	0.18	--	-5.54, 1.04
MSA → Occ Fx with CESD (c’)	-4.25	-0.15	3.60	0.24	0.14	-11.30, 2.80
**Military Sexual Harassment (MSH)**						
MSH → CESD → Occ Fx						
MSH → CESD (a)	6.07	0.39	1.61	< 0.001	0.16	2.92, 9.22
CESD → Occ Fx (b)	-0.38	-0.24	0.17	0.03	--	-0.71, -0.05
MSH → Occ Fx (c)	-3.29	-0.14	2.55	0.20	0.08	-8.29, 1.71
MSH → CESD → Occ Fx (ab)	-2.31	-0.10	1.27	0.07	--	-4.79, 0.18
MSH → Occ Fx with CESD (c’)	-1.24	-0.05	2.92	0.67	0.13	-6.97, 4.49
**Military Related Trauma (MRT)**						
MRT → CESD → Occ Fx						
MRT → CESD (a)	5.48	0.35	1.87	0.003	0.12	1.81, 9.15
CESD → Occ Fx (b)	-0.28	-0.18	0.16	0.08	--	-0.60, 0.04
MRT → Occ Fx (c)	-7.01	-0.29	2.55	0.01	0.14	-12.01, -2.00

MRT → CESD → Occ Fx (ab)	-1.55	-0.06	1.06	0.14	--	-3.62, 0.52
MRT → Occ Fx with CESD (c')	-5.70	-0.23	2.92	0.05	0.18	-11.41, 0.02
**Adult Physical Assault (APA)**						
APA → CESD → Occ Fx						
APA → CESD (a)	8.42	0.42	2.61	0.001	0.18	3.30, 13.54
CESD → Occ Fx (b)	-0.36	-0.23	0.18	0.05	--	-0.72, -0.001
APA → Occ Fx (c)	-4.43	-0.14	3.21	0.17	0.07	-10.72, 1.85
APA → CESD → Occ Fx (ab)	-3.04	-0.10	1.89	0.11	--	-6.74, 0.67
APA → Occ Fx with CESD (c')	-1.73	-0.06	3.34	0.60	0.13	-8.27, 4.81
**Adult Sexual Assault (ASA)**						
ASA → CESD → Occ Fx						
ASA → CESD (a)	5.23	0.32	2.13	0.01	0.10	1.06, 9.40
CESD → Occ Fx (b)	-0.30	-0.19	0.16	0.06	--	-0.61, 0.02
ASA → Occ Fx (c)	-6.93	-0.27	2.58	0.01	0.13	-11.99, -1.87
ASA → CESD → Occ Fx (ab)	-1.56	-0.06	1.06	0.14	--	-3.64, 0.52
ASA → Occ Fx with CESD (c')	-5.61	-0.22	2.72	0.04	0.17	-10.94, -0.28
**Child Physical Assault (CPA)**						
CPA → CESD → Occ Fx						
CPA → CESD (a)	4.63	0.24	2.35	0.05	0.06	0.02, 9.24
CESD → Occ Fx (b)	-0.37	-0.24	0.16	0.02	--	-0.68, -0.06
CPA → Occ Fx (c)	-4.33	-0.14	3.12	0.17	0.07	-10.45, 1.79
CPA → CESD → Occ Fx (ab)	-1.70	-0.06	1.14	0.14	--	-3.93, 0.53
CPA → Occ Fx with CESD (c')	-2.76	-0.09	3.44	0.42	0.14	-9.50, 3.98
**Child Sexual Assault (CSA)**						
CSA → CESD → Occ Fx						
CSA → CESD (a)	3.67	0.19	2.27	0.11	0.04	-0.78, 8.12
CESD → Occ Fx (b)	-0.40	-0.26	0.16	0.01	--	-0.71, -0.09
CSA → Occ Fx (c)	-2.03	-0.07	3.13	0.52	0.05	-8.16, 4.10
CSA → CESD → Occ Fx (ab)	-1.46	-0.05	1.03	0.16	--	-3.48, 0.56
CSA → Occ Fx with CESD (c')	-0.82	-0.03	3.32	0.81	0.13	-7.34, 5.70

International Journal of
Environmental Research and Public Health

MDPI

Note: CESD = Center for Epidemiological-Studies Depression Scale, SE = standard error, OR = odds ratio, CI = confidence interval, Occ Fx = occupational functioning. a = direct path from the trauma type to the mediator, b = direct path from the mediator to unemployment status, c = direct path from the trauma type to unemployment status, ab = indirect effect from the trauma type to unemployment status via the mediator, c' = direct path from the trauma type to unemployment status with the mediator in the model. Trauma exposure and depression symptoms were assessed at Time 1; employment status was assessed at Time 2. Models adjusted for Time 1 age, education and employment status. *N*= 90 because only women who reported being employed or volunteering in the past 30 days completed the occupational functioning measure and were included in the analyses.

International Journal of
Environmental Research and Public Health

MDPI

Table S2. Associations between trauma types and occupational functioning, with PTSD symptoms as a mediator.

Path	B	β	SE	*p*	R^2	95% CI
**Military Sexual Trauma (MST)**						
MST → PCL → Occ Fx						
MST → PCL (a)	10.38	0.38	2.77	< 0.001	0.14	4.96, 15.80
PCL → Occ Fx (b)	-0.39	-0.45	0.09	< 0.001	--	-0.56, -0.21
MST → Occ Fx (c)	-2.95	-0.12	2.51	0.24	0.07	-7.87, 1.98
MST → PCL → Occ Fx (ab)	-4.03	-0.17	1.27	0.002	--	-6.52, -1.54
MST → Occ Fx with PCL (c')	1.06	0.05	2.63	0.69	0.23	-4.11, 6.22
**Military Sexual Assault (MSA)**						
MSA → PCL → Occ Fx						
MSA → PCL (a)	16.81	0.53	4.16	< 0.001	0.28	8.66, 24.97
PCL → Occ Fx (b)	-0.37	-0.42	0.10	< 0.001	--	-0.56, -0.17
MSA → Occ Fx (c)	-6.56	-0.24	2.82	0.02	0.12	-12.08, -1.04
MSA → PCL → Occ Fx (ab)	-6.14	-0.22	2.23	0.01	--	-10.52, -1.77
MSA → Occ Fx with PCL (c')	-0.44	-0.02	3.53	0.90	0.23	-7.37, 6.49
**Military Sexual Harassment (MSH)**						
MSH → PCL → Occ Fx						
MSH → PCL (a)	10.73	0.39	2.78	< 0.001	0.15	5.27, 16.18
PCL → Occ Fx (b)	-0.39	-0.45	0.09	< 0.001	--	-0.57, -0.21
MSH → Occ Fx (c)	-3.29	-0.14	2.55	0.20	0.08	-8.29, 1.71
MSH → PCL → Occ Fx (ab)	-4.16	-0.18	1.30	0.001	--	-6.71, -1.61
MSH → Occ Fx with PCL (c')	0.98	0.04	2.72	0.72	0.23	-4.34, 6.30
**Military Related Trauma (MRT)**						
MRT → PCL → Occ Fx						
MRT → PCL (a)	11.89	0.43	3.20	< 0.001	0.18	5.62, 18.15
PCL → Occ Fx (b)	-0.33	-0.38	0.09	< 0.001	--	-0.50, -0.16
MRT → Occ Fx (c)	-7.01	-0.29	2.55	0.01	0.14	-12.01, -2.00

MRT → PCL → Occ Fx (ab)	-3.88	-0.16	1.34	0.004	--	-6.50, -1.26
MRT → Occ Fx with PCL (c')	-3.45	-0.14	2.66	0.19	0.27	-8.66, 1.76
**Adult Physical Assault (APA)**						
APA → PCL → Occ Fx						
APA → PCL (a)	19.01	0.53	4.59	< 0.001	0.28	10.02, 28.0
PCL → Occ Fx (b)	-0.42	-0.48	0.10	< 0.001	--	-0.62, -0.21
APA → Occ Fx (c)	-4.43	-0.14	3.21	0.17	0.07	-10.72, 1.85
APA → PCL → Occ Fx (ab)	-7.89	-0.26	2.75	0.004	--	-13.27, -2.50
APA → Occ Fx with PCL (c')	3.09	0.10	3.37	0.36	0.24	-3.50, 9.69
**Adult Sexual Assault (ASA)**						
ASA → PCL → Occ Fx						
ASA → PCL (a)	12.10	0.42	3.48	0.001	0.17	5.28, 18.92
PCL → Occ Fx (b)	-0.33	-0.38	0.09	< 0.001	--	-0.50, -0.16
ASA → Occ Fx (c)	-6.93	-0.27	2.58	0.01	0.13	-11.99, -1.87
ASA → PCL → Occ Fx (ab)	-4.02	-0.16	1.50	0.01	--	-6.95, -1.08
ASA → Occ Fx with PCL (c')	-3.08	-0.12	2.72	0.26	0.25	-8.41, 2.24
**Child Physical Assault (CPA)**						
CPA → PCL → Occ Fx						
CPA → PCL (a)	11.92	0.34	4.67	0.01	0.12	2.76, 21.08
PCL → Occ Fx (b)	-0.38	-0.44	0.08	< 0.001	--	-0.54, -0.21
CPA → Occ Fx (c)	-4.33	-0.14	3.12	0.17	0.07	-10.45, 1.79
CPA → PCL → Occ Fx (ab)	-4.48	-0.15	2.05	0.03	--	-8.51, -0.46
CPA → Occ Fx with PCL (c')	-0.03	-0.001	3.26	0.99	0.24	-6.41, 6.35
**Child Sexual Assault (CSA)**						
CSA → PCL → Occ Fx						
CSA → PCL (a)	4.14	0.12	3.60	0.25	0.02	-2.9, 11.19
PCL → Occ Fx (b)	-0.38	-0.44	0.08	< 0.001	--	-0.53, -0.23
CSA → Occ Fx (c)	-2.03	-0.07	3.13	0.52	0.05	-8.16, 4.10
CSA → PCL → Occ Fx (ab)	-1.57	-0.05	1.44	0.28	--	-4.39, 1.25
CSA → Occ Fx with PCL (c')	-0.58	-0.02	2.85	0.84	0.25	-6. 17, 5.01

International Journal of
Environmental Research and Public Health
MDPI

Note: PCL = PTSD Check List, Occ Fx = occupational functioning, SE = standard error, OR = Odds Ratio, CI = Confidence Interval. a = direct path from the trauma type to the mediator, b = direct path from the mediator to unemployment status, c = direct path from the trauma type to unemployment status, ab = indirect effect from the trauma type to unemployment status via the mediator, c' = direct path from the trauma type to unemployment status with the mediator in the model. Trauma exposure and PTSD symptoms were assessed at Time 1; employment status was assessed at Time 2. Models adjusted for Time 1 age, education and employment status. *N*= 90 because only women who reported being employed or volunteering in the past 30 days completed the occupational functioning measure and were included in the analyses.
